# Retraction: Blockage of transdifferentiation from fibroblast to myofibroblast in experimental ovarian cancer models

**DOI:** 10.1186/1476-4598-8-84

**Published:** 2009-10-14

**Authors:** Qin Yao, Xun Qu, Qifeng Yang, David A Good, Shuzhen Dai, Beihua Kong, Ming Q Wei

**Affiliations:** 1 Division of Molecular and Gene Therapies, Griffith Institute for Health and Medical Research, School of Medical Science, Griffith University, Gold Coast campus, Southport, Qld 4222, Australia; 2 Department of Obstetrics and Gynaecology, Qilu Hospital, Shandong University, Ji'nan 250012, Shandong, PR China; 3 Department of Obstetrics and Gynaecology, Affiliated Hospital of Qingdao University, Qing'dao 266003, Shandong, PR China; 4 Institute of Basic Medical Sciences, Qilu Hospital, Shandong University, Ji'nan 250012, Shandong, PR China

## Retraction

This article [1] was submitted to *Molecular Cancer *following the acceptance of an article in *Oncology Reports *[2], published in September 2009. The two articles were produced from the same data and as a result of miscommunication between authors contained extensive overlap. As such the authors would like to retract the *Molecular Cancer *article. The authors would like to apologise for any inconvenience this may have caused to the editorial staff and readers.
